# COX-2 in the neurodegenerative process of Parkinson's disease

**DOI:** 10.1002/biof.1035

**Published:** 2012-07-23

**Authors:** Peter Teismann

**Affiliations:** School of Medical Sciences, College of Life Sciences and Medicine, University of AberdeenAberdeen AB25 2ZD, Scotland, UK

**Keywords:** cyclooxygenase-2, Parkinson's disease, neuroinflammation, neurodegeneration

## Abstract

The enzyme cyclooxygenase-2 (COX-2), responsible for the first committed step in the synthesis of several important mediators which are involved in both initiation and resolution of inflammation, and the subsequent generation of prostaglandins (PGs) upon activation has been shown to participate in the neurodegenerative processes of a variety of diseases. This review looks particular at the role of COX-2 in the pathogenesis of Parkinson's disease, involving the generation of PGs and the role of the two different parts of the cyclooxygenase—cyclooxygenase and peroxidase activity.

## 1. Introduction

Neuroinflammation has long been implicated in the pathogenesis of a variety of neurodegenerative diseases, as Alzheimer's disease, amyotrophic lateral sclerosis, and Parkinson's disease (PD) [1]. Markers of inflammation, particularly an increase in activated microglia, have been reported in PD [2, 3]. Furthermore, an increased expression of cyclooxygenase-2 (COX-2) has long been associated with the disease. Cyclooxygenase (COX) is the main enzyme responsible for the conversion of arachidonic acid into prostaglandin (PG) H2, which is the main precursor of the different PGs, but in particular PGE2. COX comes in three different isoforms: 1) COX-1, which is in general constitutively expressed and present in many cell types. 2) COX-2, which in general is expressed on a wide array of stimuli, in particular in response to *N*-methyl-d-aspartate (NMDA)—dependent synaptic activity [4]. Furthermore, a low level of COX-2 expression can be found in the central nervous system [5]. 3) COX-3, made from the COX-1 gene, was first described in 2002 [6]. It has been linked to the action of acetaminophen (paracetamol), as the drug possesses weak COX-1 and COX-2 inhibitory effects, but potent antipyretic and analgesic activity. COX-3 seems to be constitutively expressed, and is either an enzyme of its own, derived by the COX-1 gene, or a variant of COX-1 (or even COX-2) (for a discussion on the issue see ref.^7^). It has to be mentioned that, after the initial enthusiasm for the discovery, COX-3 functional role in human brain remains, at present, uncertain [8, 9].

All Cox enzymes catalyze the formation of PGs from arachidonic acid. In a first cyclooxygenase reaction, arachidonic acid and two O_2_ molecules are converted to form PGG_2_. In the second, peroxidase reaction step PGG_2_ is reduced by two electrons to form PGH_2_ [10]. The main differences between COX-1 and COX-2 in peroxidase activity are determined by two facts: first of all by the kinetics involved: Intermediates appearing in the second step of PGH2 generation are far more rapidly formed by COX-2 than COX-1. Second: COX-1 utilizes a two-electron reduction of hydroperoxidase substrates whereas in the case of COX-2 it is to ∼40% one-electron reduction [11]. The one electron reduction has long been implicated to lead to the leakage of electrons, which in turn could react with cellular oxygen to form reactive oxygen species [12, 13]. Interestingly enough, it has been reported that only carbon-centered radicals are generated in the COX-2/arachidonic acid system and are responsible for the generation of oxidative stress [14].

Based on the hypothesis that peroxidase activation of COX-2 can be detrimental the role of COX-2 peroxidase as well as COX-2 cyclooxygenase activity has been investigated in detail. A study using adenoviral overexpression of COX-2 with a mutation in the peroxidase site of COX-2 led to similar susceptibility to hypoxia compared with those cells overexpressing normal COX-2 [15] In contrast, a mutation in the cyclooxygenase site led to a protective effect against hypoxia. The authors hypothesize that the protective effect is caused by the inability of arachidonic acid to bind to the modified COX-2 and thus the enzyme cannot generate PGs [15, 16]. Recently, a new mouse model for specific cyclooxygenase ablation, leaving peroxidase activity intact, has been generated [17], modeling the specific COX-2 inhibition of newer COX-2 inhibitors such as celecoxib and rofecoxib. The authors report that COX-1 and COX-2 can form heterodimers, which are capable of producing PGs. Unfortunately it seems that current techniques will not be able to distinguish between the effect of specific COX-2 inhibition on COX-2 homodimers or COX-1-COX-2 heterodimers [17]. Still the model provides a new tool in dissecting the different COX-2 mechanisms to generate new substances, which in the end might provide the beneficial effect as seen in disease models, without the sometimes severe side-effects.

## 2. COX-2 in models of Parkinson's disease

The main neurotoxin models to study PD are based on the administration of a neurotoxin as 1-methyl-4-phenyl-1,2,3,6-tetrahydropyridine (MPTP) or 6-hydroxydoamine (6-OHDA) (a review on the models can be found in ref.^18^). Inhibition of COX-2 by acetylsalicylic acid and salicylate provided neuroprotection in the MPTP-model [19, 20], whereas diclofenac showed no neuroprotective effect. The later could be dependent on its failure to penetrate the blood–brain barrier, as on the other hand meloxicam was able to protect against MPTP-induced toxicity [20]. Using COX-2 deficient mice, a significant role for COX-2 in the MPTP-model was confirmed, as these mice showed significant protection against MPTP-induced neurodegeneration [21, 22]. COX-2 was mainly expressed in dopaminergic neurons after MPTP, which is partially in contrast to another publication who describes a more abundant expression of COX-2 in microglia [23]. Differences could be related to technical differences; nevertheless, both studies agree that neurons compose the majority of COX-2 positive cells in PD.

Interestingly enough, microglia expression was not reduced after MPTP in COX-2 deficient mice, despite the fact of a reduction in cellular loss. This could be due to the fact, that a very harsh MPTP regimen was used (4 × 20 mg//kg i.p. 2 h apart), and not the “sub-acute” or chronic model (30 mg/kg i.p. over five consecutive days) of the disease, leading to a more progressive invasion of microglia. Besides, other factors which contribute to the cellular demise as inducible nitric oxide (iNOS) were not affected. Further investigation showed that dopamine-quinone, a by-product generated by COX-2 activity was highly upregulated. In turn, mice which received the COX-2 inhibitor rofecoxib did not show any attenuation of dopamine quinone expression after MPTP when compared to saline-treated control animals. As described previously, COX-2 can lead to the oxidation of dopamine to form dopamine-quinone [24], which in turn is highly reactive. Dopamine-quinone can react with cysteinyl residues in proteins, leading to protein transformation and subsequently to alteration of protein function. This in turn can have led to the cell death observed after MPTP, and thus be one explanation for the protective effect of COX-2 ablation [21].

A second pathway by which COX-2 possibly leads to cellular demise after MPTP is by increasing the levels of PGE_2_. PGE_2_ levels were only slightly affected by COX-2 ablation after MPTP administration in our studies, but again, this could be due to the fact, that a “harsh” regimen of MPTP administration was used. Increased turnover of PGE_2_ can lead to elevated levels of reactive oxygen species [25] and, PGE_2_ can lead to the activation of astrocytes [26].

Additionally, PGE_2_ can interact with different EP receptors, thus promoting neurodegeneration (a full review of the four different PG E receptors can be found in ref.^27^). Of the receptors described, only the EP2 receptor has been studied in a model of PD. Microglial activation and associated neurotoxicity seems to be mediated by EP2 [28], as EP2 deficient mice showed protection against MPTP-induced toxicity. Also, EP2^−/−^ microglia enhanced the clearance of α-synuclein in tissue sections obtained from patients with Lewy body disease. On the other hand, the EP2 receptor protects against 6-OHDA toxicity in a cell culture model [29]. One has to keep in mind that the later study uses cell culture, lacking microglia, and EP2 seems to act via microglia. Thus, it is questionable if the later study indeed describes reliably an effect which could be reproduced *in vivo*. It is also described that lipopolysaccharide (LPS) does not induce secondary neurotoxicity in conditioned medium from EP2^−/−^ microglia, suggesting an important role for EP2 in inflammatory reactions and LPS-mediated neurotoxicity [30].

Looking at EP1 it becomes clear that this receptor might also contribute to PGE_2_-mediated toxicity. It has been described to make neurons more susceptible to oxidative stress in a cell culture model of PD [31]. EP1 receptors seem to be the main pathway by which COX-2 mediates neurotoxicity through disruption of Ca^2+^ homeostasis [32]. Another pathway by which EP1 could mediate toxicity is by reducing energy levels, as activation of the EP1 receptor has been shown to lead to a long duration oxygen-glucose deprivation [33].

Additionally, the expression of pro-inflammatory cytokines such as IL-6 is regulated by PGE_2_ in various cell-types like macrophages and astrocytes [34–36]. Selective antibody neutralization of PGE_2_ inhibits IL-6 production, hyperalgesia, and the inflammatory process in a model of carrageen-induced paw-inflammation [37]. Whether this pathway—COX-2—IL-6 also plays a role in PD remains to be shown as studies investigating the role of COX-2 and IL-6 only have shown a parallel increase [38, 39].

Taken together we can say that COX-2 plays a fundamental part in the pathogenesis of PD, and if only as a propagator of the disease. Inhibition of COX-2 remains a valuable target as a potential neuroprotective treatment strategy aimed at slowing or halting the progression of the disease.
